# Somatic mutation correlation with lymph node metastasis and prognosis in T1/2 stage colorectal cancer patients: A propensity score matching analysis

**DOI:** 10.1002/ctm2.70179

**Published:** 2025-01-07

**Authors:** Junwei Tang, Yue Zhang, Dongjian Ji, Lu Wang, Han Zhuo, Yaping Wang, Yueming Sun

**Affiliations:** ^1^ Department of General Surgery Colorectal Institute of Nanjing Medical University The First Affiliated Hospital of Nanjing Medical University Nanjing China; ^2^ Hepatobiliary Center The First Affiliated Hospital of Nanjing Medical University Nanjing China; ^3^ Department of Hematology and Oncology Children's Hospital of Nanjing Medical University Nanjing Medical University Nanjing China

1

Dear Editor,

Our study provides a comprehensive analysis of the correlation between specific somatic mutations and the lymph node metastasis in colorectal cancer (CRC) patients with T1/2 stage, addressing a significant gap in the early‐stage prognosis and individualised treatment of CRC. By leveraging next‐generation sequencing (NGS) and clinical data, we identified key mutations and pathological characteristics that can serve as robust predictors of lymph node metastasis, thereby enhancing clinicians' ability to stratify T1/2 CRC patients based on metastatic risk.

CRC remains a prevalent malignancy worldwide, with early‐stage cases, particularly T1/2 stages, generally associated with favourable outcomes.^1^ However, when lymph node metastasis is present, the prognosis worsens significantly.^2^ Lymph node involvement in early‐stage CRC is a strong indicator of potential distant metastasis and recurrence, highlighting the urgency of effective preoperative risk assessment in T1/2 patients.^3^ Existing imaging techniques, such as enhanced computed tomography and magnetic resonance imaging, offer limited sensitivity and specificity for lymph node assessment, especially in early stages where inflammation or small metastatic nodes may escape detection.^4^, ^5^ Therefore, identifying molecular markers that can predict lymph node involvement with high accuracy is paramount to guiding treatment decisions in early‐stage CRC. Our study contributes to this goal by identifying specific genetic mutations and clinical features associated with metastatic risk, thus enabling a more refined preoperative evaluation of T1/2 stage CRC patients.

We conducted a retrospective cohort study including 212 T1/2 CRC patients (Figure S1), who were categorised based on lymph node involvement into T1/2N+ and T1/2N‒ groups and matched using propensity score analysis (Figure 1 and Table S1). Our NGS data showed that mutations in four genes (LRP1B, KMT2B, TSC2 and BRAF) were significantly more frequent in the T1/2N+ group (Figure 2), and the presence of any of these mutations correlated with reduced overall survival (Figure S2). Among the four, BRAF mutations have been widely recognised in literature as a poor prognostic factor in advanced CRC,^6^ but their impact in early stages has remained largely unexplored until now. Additionally, LRP1B, KMT2B and TSC2 mutations appear to be novel findings in the context of early‐stage lymph node metastasis in CRC, indicating that they could potentially be unique markers for predicting outcomes in T1/2 patients (Figure 3). Our analysis also showed that patients with LRP1B mutations had particularly poor survival outcomes, with a median survival of 22.2 months.

**FIGURE 1:**
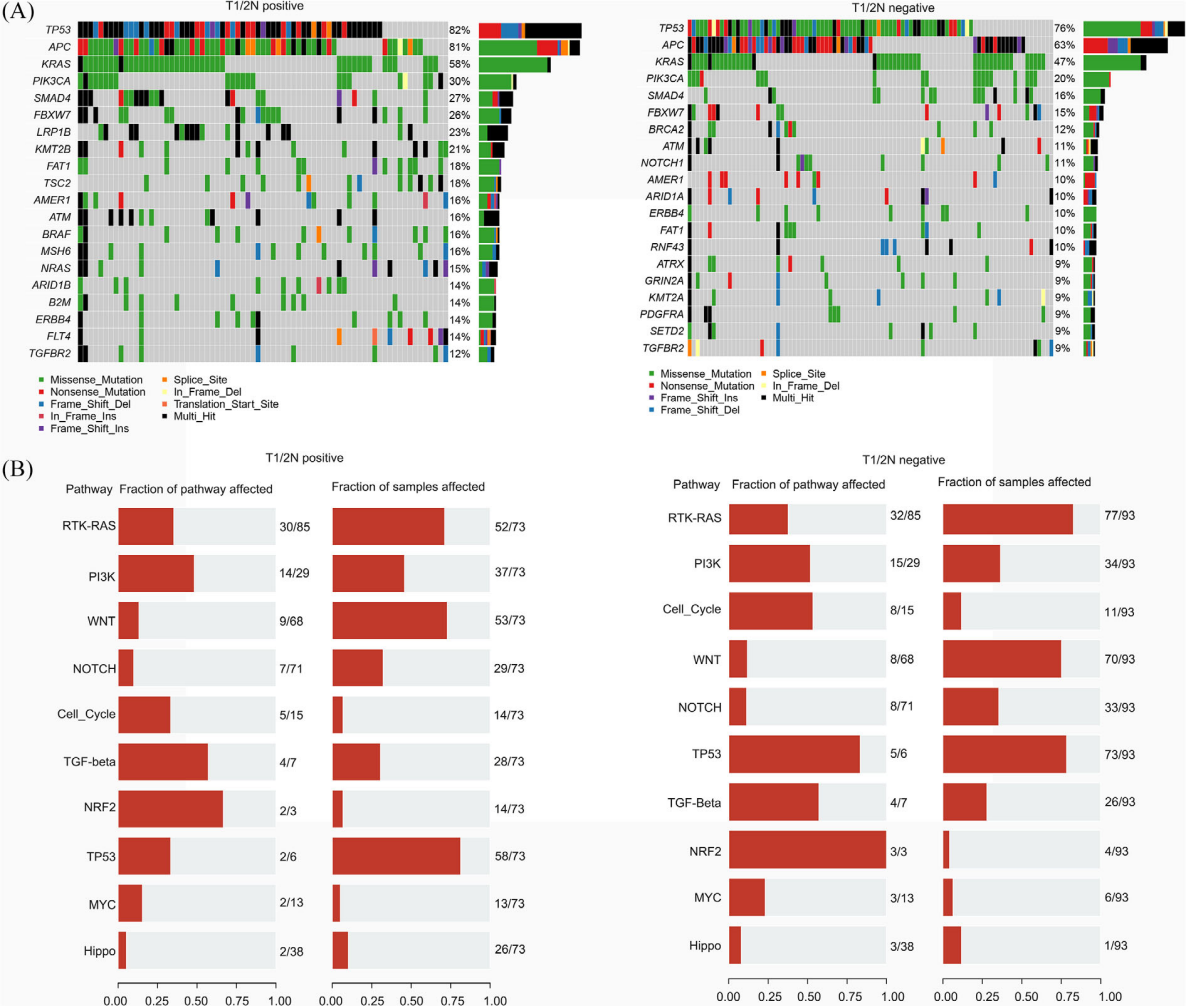
Mutation landscape difference in T1/2N+ and T1/2N‒ colorectal cancer patients. (A) The detailed mutation frequency of genes in T1/2N+ and T1/2N‒ colorectal cancer patients. The types of mutations are coloured differentially and labelled. (B) The fraction of mutated genes involved in each pathway is displayed in two groups.

**FIGURE 2:**
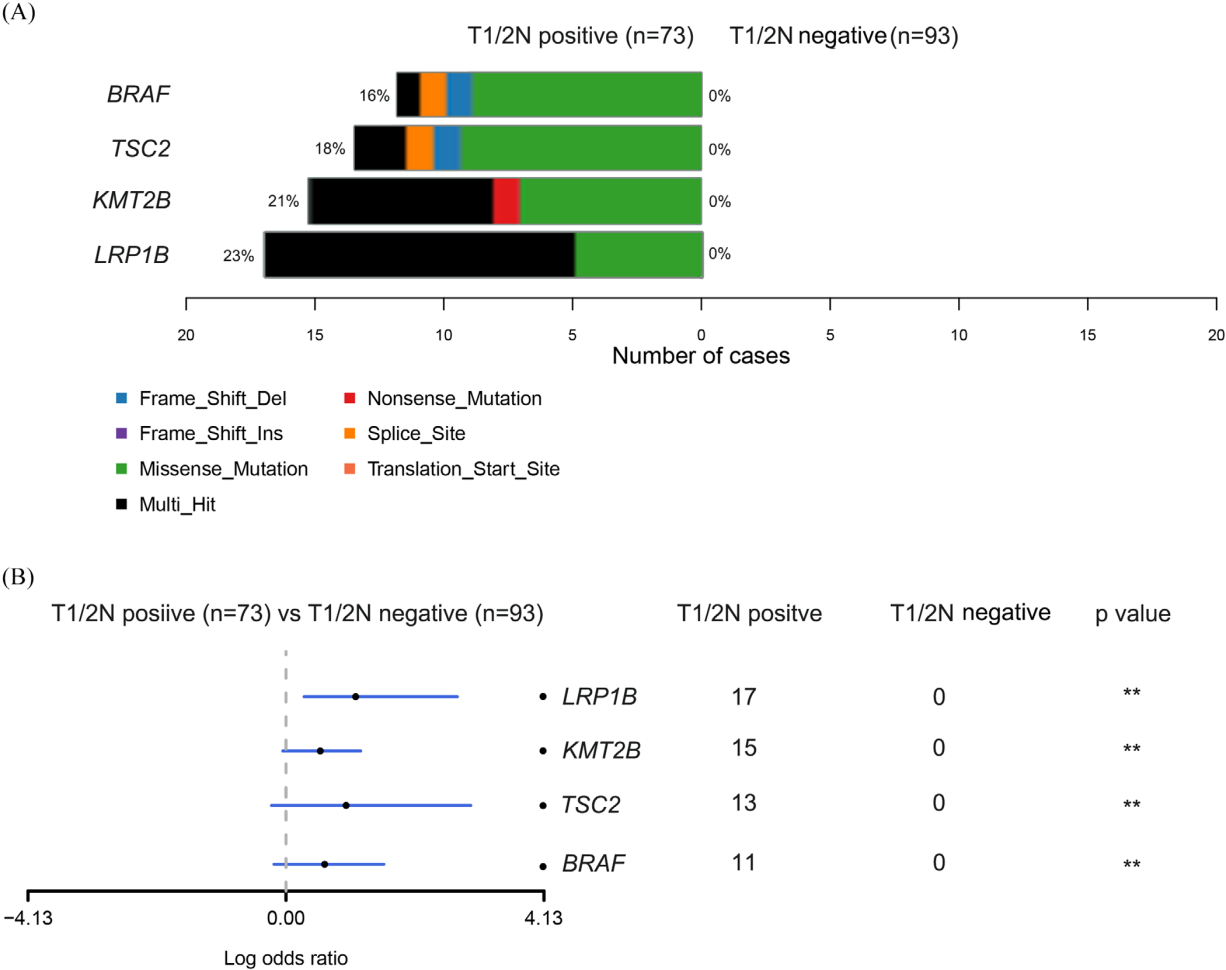
Somatic mutation difference in MSS T1/2N+ and T1/2N‒ colorectal cancer patients. (A) The detailed mutation frequency of genes in T1/2N+ and T1/2N‒ colorectal cancer patients. The types of mutations are coloured differentially and labelled. (B) The mutation frequency difference was presented in forest maps with odds ratios (ORs). MSS, Microsatellite Stability.

**FIGURE 3:**
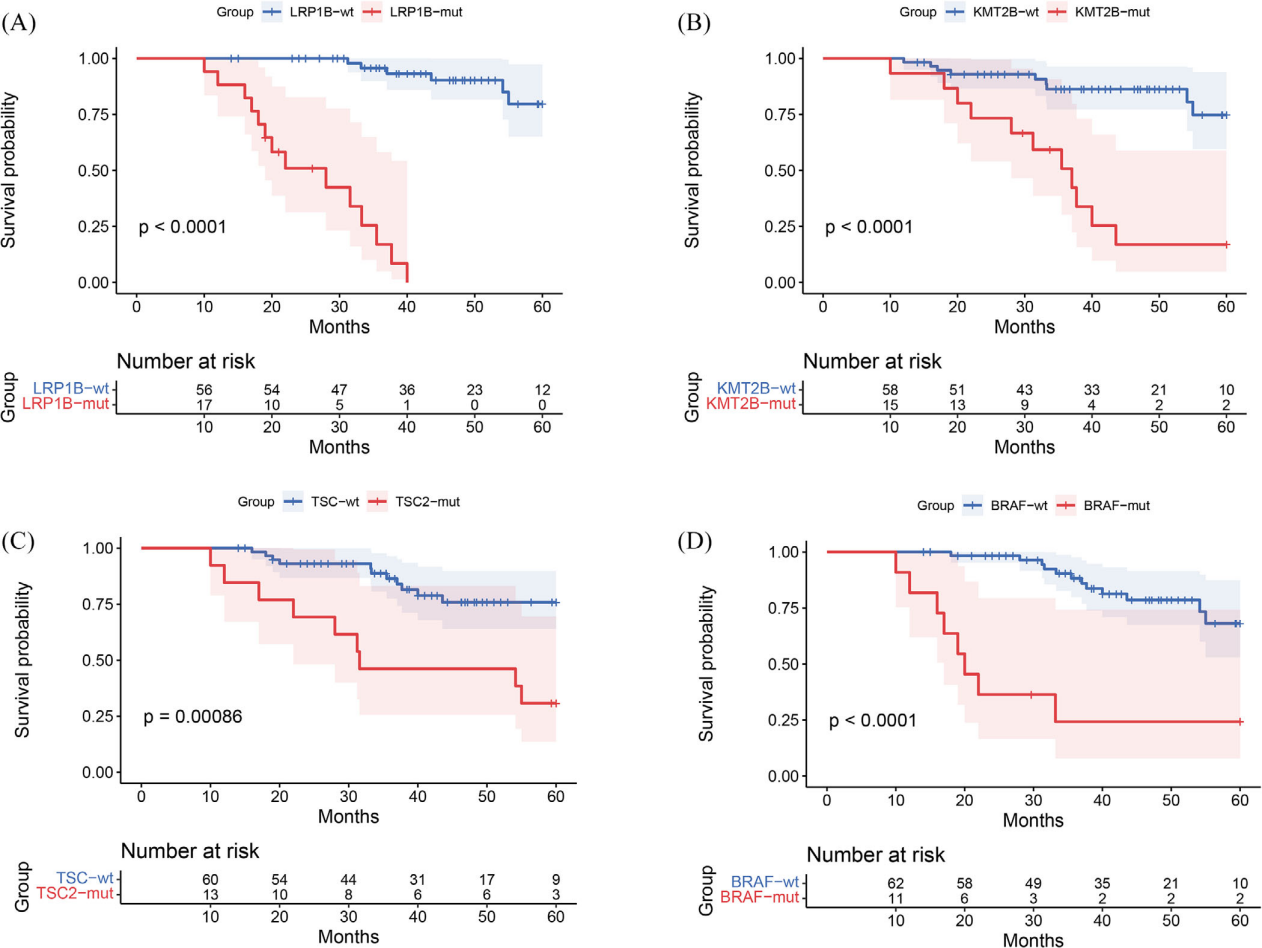
Stratified overall survival analysis of T1/2N+ colorectal cancer patients based on specific mutations. Patients were stratified based on the presence or absence of alterations in the following genes. (A) LRP1B (HR = 2.86, 95% confidence interval [CI]: [1.74, 4.70]), (B) KMT2B (HR = 1.95, 95% CI: [1.18, 3.21]), (C) TSC2 (HR = 2.34, 95% CI: [1.34, 4.08]) and (D) BRAF (HR = 3.12, 95% CI: [1.67, 5.84]). The number at risk at each time point is indicated in the adjacent risk table. HR, Hazard Ratio.

In addition to these molecular findings, there was no significant difference in the distribution of MSI status and TMB between the two groups; however, we observed significant differences in clinical characteristics between T1/2N+ and T1/2N‒ patients. T1/2N+ patients had higher rates of lymphovascular invasion, poor tumour differentiation and aggressive histological subtypes. The presence of lymphovascular invasion, an established risk factor for metastasis, was notably higher in T1/2N+ patients (66 .0% vs. 51.9%, *p* = .036). Poorly differentiated tumours were also more common in T1/2N+ patients, with the proportion being nearly double that of the T1/2N‒ group (32.1% vs. 16.1%, *p* = .019). These pathological characteristics underscore the heterogeneity within T1/2 stage CRC and the importance of integrating clinical and molecular data to predict metastatic risk accurately. Histological type also played a role, with higher proportions of mucinous and signet‐ring cell carcinomas observed in the T1/2N+ group, both of which are histologically aggressive subtypes associated with poor prognosis (Table S2).

Given the clinical importance of preoperative assessment for lymph node metastasis, we developed a predictive nomogram model incorporating the identified mutations, lymphovascular invasion, tumour differentiation and histological type (Figure 4A and Table S3). This nomogram provides a scoring system where each risk factor contributes to an overall score that correlates with the probability of lymph node metastasis. Furthermore, this model exhibits high predictive accuracy, with an area under the curve of 81.3%, underscoring its reliability in evaluating metastatic risk in early‐stage CRC patients (Figure 4B). Clinicians can leverage this tool to make more informed decisions about surgical strategies, including the extent of lymph node dissection, and to assess the necessity of postoperative adjuvant therapies. By stratifying patients based on their risk scores, the nomogram allows for a more personalised treatment strategy, potentially improving survival outcomes and reducing the need for overtreatment in low‐risk cases.

**FIGURE 4:**
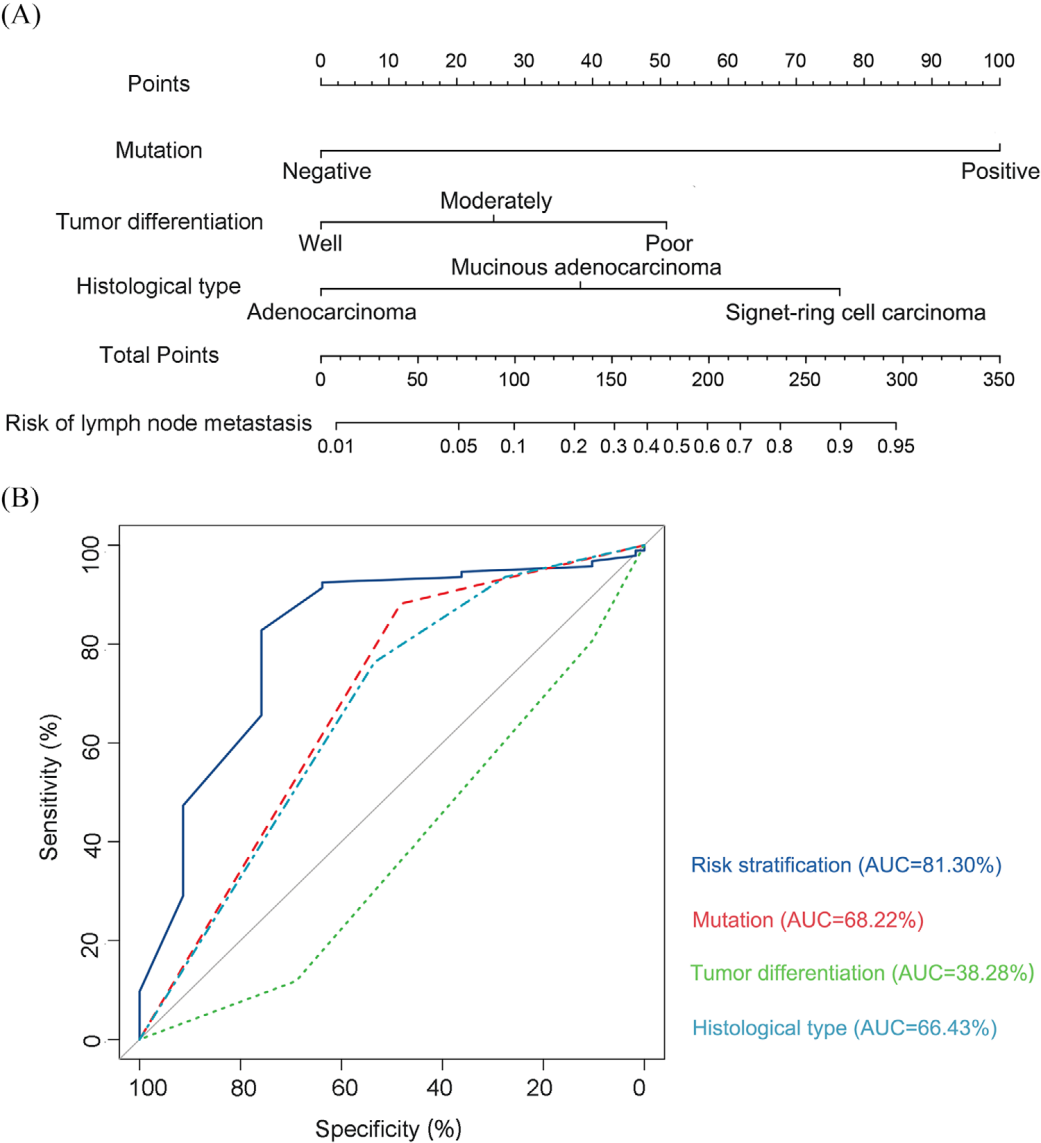
Clinical and molecular characteristics as prediction model for lymph node metastasis. (A) Nomogram representing the probability of lymph node metastasis risk. For clinical purposes, scores for each covariate are summed, and the total score is depicted on the total points axis. (B) Receiver operating characteristic curves for clinical and molecular characteristic in cohort.

Our study also sheds light on the prognostic value of these specific genetic mutations in early‐stage CRC. For instance, BRAF mutations are already associated with poor outcomes in CRC, particularly in metastatic cases. Our study confirms this trend even in early‐stage CRC, suggesting that BRAF mutations could serve as a marker for aggressive disease progression, even before metastasis is evident. Similarly, KMT2B and TSC2 mutations, although not well‐studied in CRC, have been implicated in other malignancies for their roles in enhancing tumour aggression and stemness,^7^, ^8^ further supporting their inclusion as high‐risk markers in our model. The mutation of LRP1B, typically associated with poor prognosis in other cancer types,^9^ also emerged as a significant indicator of poor survival in our cohort, underscoring the potential importance of this mutation in the early metastatic pathway of CRC. Our findings emphasise the need for a multidimensional approach in the management of T1/2 stage CRC patients. The integration of molecular and clinical markers in a predictive model provides a more accurate assessment of lymph node metastasis risk, which is crucial for guiding treatment decisions in early‐stage CRC. Current clinical practice primarily reserves targeted therapies for advanced stages, but our findings suggest that high‐risk T1/2 patients may also benefit from targeted approaches aimed at specific genetic mutations, which could ultimately improve their prognosis.

Our study also has several limitations. First, the retrospective nature of the study design limits the level of evidence provided. Future prospective studies are needed to provide more robust and comprehensive results. Moreover, the application of this predictive model requires further precise analysis to establish specific weights, which will be crucial for guiding subsequent treatment decisions.

In conclusion, our study identifies mutations in LRP1B, KMT2B, TSC2 and BRAF as significant prognostic markers in T1/2 CRC, along with critical clinical features such as lymphovascular invasion, poor tumour differentiation and aggressive histological types. By combining these factors into a predictive nomogram model, we provide a tool that enables accurate preoperative assessment of lymph node metastasis risk, facilitating a more personalised treatment approach for early‐stage CRC patients. Our comprehensive approach represents a significant step towards optimising outcomes and enhancing the quality of care for T1/2 stage CRC patients.

## AUTHOR CONTRIBUTIONS

*Conceptualisation, methodology, software*: Priya Singh. *Data curation and writing—original draft preparation*: Junwei Tang. *Methodology, visualisation and investigation*: Yue Zhang. *Supervision*: Dongjian Ji. *Software and validation*: Lu Wang. *Methodology, writing—reviewing and editing, and project administration*: Han Zhuo. *Methodology, software Priya Singh and project administration*: Yaping Wang. *Supervision, project administration and funding acquisition*: Yueming Sun.

## CONFLICT OF INTEREST STATEMENT

The authors declare they have no conflicts of interest.

## FUNDING INFORMATION

This work was supported by the National Natural Science Foundation (82273406, 82473049 and 82304221), Basic Research Program of Jiangsu Province (BK20221415 and BK20230730), Jiangsu Key Medical: Discipline (General Surgery; grant no. ZDXK202222) and China Postdoctoral Science Foundation (2022M721679).

## ETHICS STATEMENT

This cohort study was approved by institutional review boards of the First Affiliated Hospital of Nanjing Medical University. Informed consent was obtained from all patients.

## Supporting information



Supporting Information

Supporting Information

## Data Availability

The data sets used in the current study are available from the corresponding author upon reasonable request.
