# Supplementary material for: Somatic mutation correlation with lymph node metastasis and prognosis in T1/2 stage colorectal cancer patients: A propensity score matching analysis
Source: Clin Transl Med. 2025 Jan 7;15(1):e70179. doi: 10.1002/ctm2.70179 (PMC11705726; doi:10.1002/ctm2.70179)
Supplement: Supplementary file 1 — Supporting Information [file CTM2-15-e70179-s002.docx]

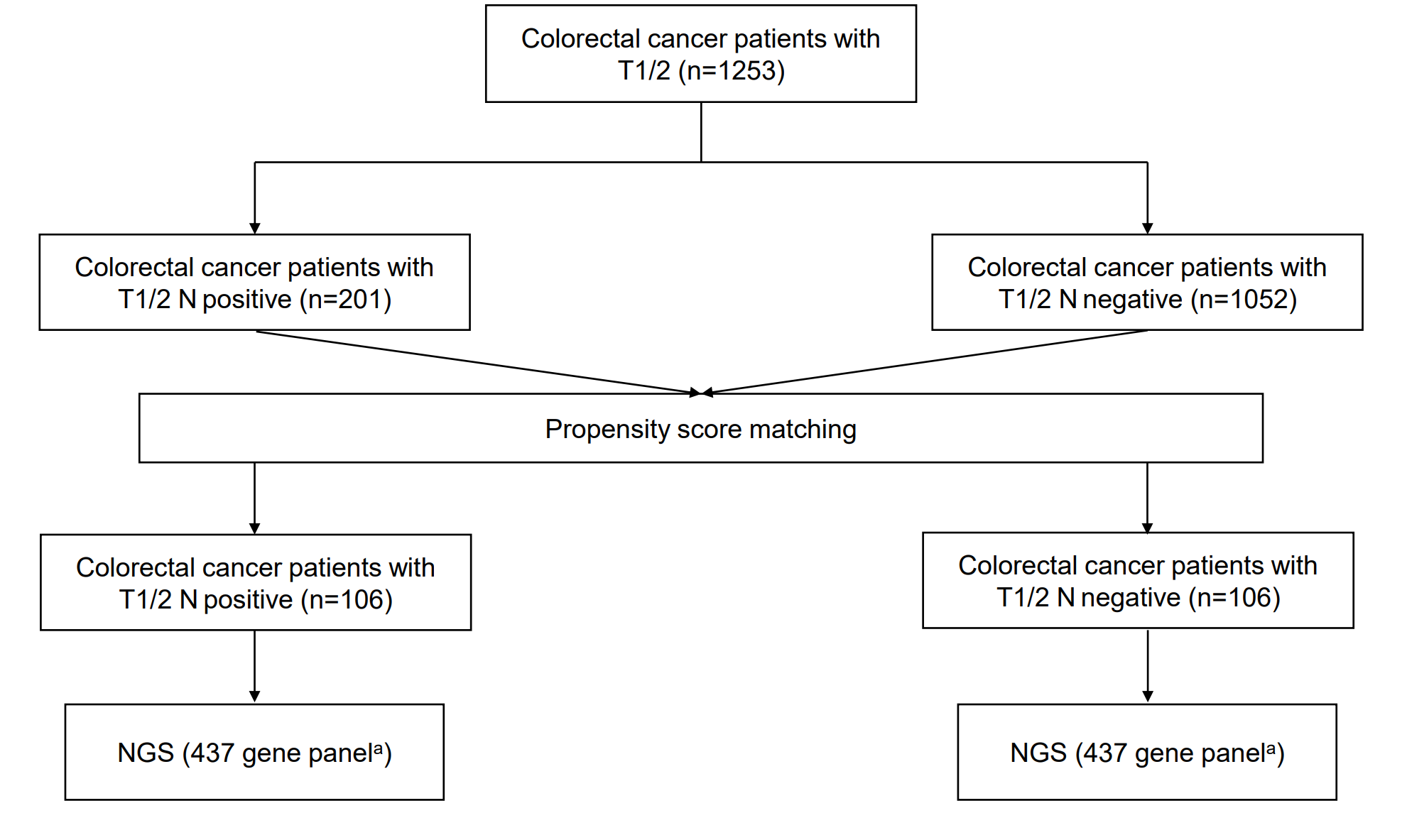


**FigureS1 Flowchart**

NGS, next-generation sequencing.

a The panel contains 437 genes for sequencing (GENESEEQPRIME™)


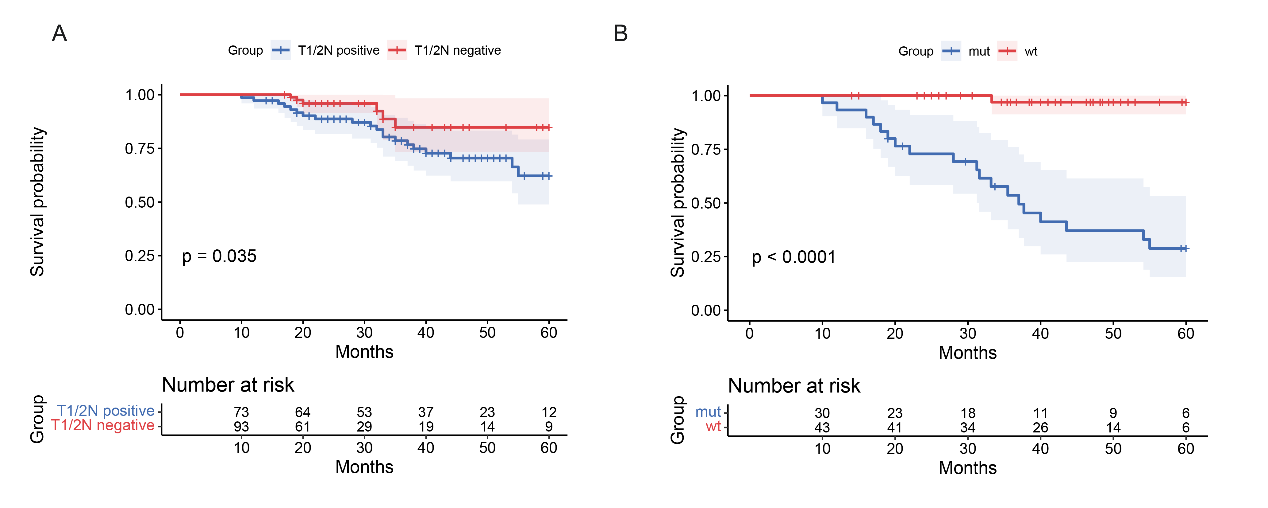


**FigureS2** **Prognosis of T1/2 stage colorectal cancer based on N stage and mutation stage.**

**A** Kaplan-Meier plots showing overall survival between lymph node status of T1/2 stage of patients with colorectal cancer. **B** Kaplan-Meier plots showing overall survival based on mutation status of T1/2 stage of patients with colorectal cancer. The number at risk was presented below, mut, mutation; wt, wild type.

**Table S1. Baseline characteristics of the colorectal cancer patients of T1/2 stage and matched cohorts.**

| **Characteristics** | **Patients No. (%) unmatched** | | | **Patients No. (%) matched** | | |
| --- | --- | --- | --- | --- | --- | --- |
|  | **T1/2N positive**  **(n=201)** | **T1/2N**  **negative**  **(n=1052)** | ***P* value** | **T1/2N positive**  **(n=106)** | **T1/2N**  **negative**  **(n=106)** | ***P* value** |
| **Age** |  |  |  |  |  |  |
| Mean (SD)^a^ | 60.03(11.12) | 64.9(9.77) | 0.032 | 63.22(10.78) | 63.41(9.81) | 0.171 |
| **Sex** |  |  |  |  |  |  |
| Male | 118(58.7) | 499(47.4) | 0.003 | 69(65.1) | 66(62.3) | 0.668 |
| Female | 83(41.3) | 553(52.6) |  | 37(34.9) | 40(37.7) |  |
| **Tumor location** |  |  |  |  |  |  |
| Ileocecal junction | 37(18.4) | 211(20.1) | 0.129 | 18(17.0) | 11(10.4) | 0.599 |
| Ascending | 33(16.4) | 215(20.4) |  | 17(16.0) | 15(14.1) |  |
| Transverse | 16(7.9) | 101(9.6) |  | 8(7.5) | 6(5.7) |  |
| Descending | 20(10.0) | 116(11.0) |  | 7(6.6) | 6(5.7) |  |
| Sigmoid | 32(16.0) | 277(16.8) |  | 20(18.9) | 21(19.8) |  |
| Rectal | 63(31.3) | 232(22.1) |  | 36(34.0) | 47(44.3) |  |
| **Tumor diameter** |  |  |  |  |  |  |
| Mean (SD) (cm) | 1.8(1.3) | 2.2 (1.5) | 0.016 | 1.7(1.0) | 1.8(0.9) | 0.129 |
| **CEA (ng/ml)** |  |  |  |  |  |  |
| >5 | 45(22.4) | 491(46.7) | <0.001 | 35(33.0) | 41(38.7) | 0.390 |
| ≤5 | 156(77.6) | 561(53.3) |  | 71(67.0) | 65(61.3) |  |
| **T stage** |  |  |  |  |  |  |
| T1 | 119(59.2) | 733(69.7) | 0.004 | 83(78.3) | 77(72.6) | 0.338 |
| T2 | 82(40.8) | 319(30.3) |  | 23(21.7) | 29(27.4) |  |

a: Mean (Standard Deviation)

**Table S2. Clinical and molecular characteristics of colorectal cancer patients of T1/2 stage.**

| **Characteristics** | **Patients No. (%)** | | |
| --- | --- | --- | --- |
|  | **T1/2N^+^**  **(n=106)** | **T1/2N^-^**  **(n=106)** | ***P* value** |
| **MSI status** |  |  |  |
| MSI-H | 8 (7.5) | 11(10.4) | 0.471 |
| MSI-L/MSS | 98(92.5) | 95(89.6) |  |
| **TMB (Mutations/Mb)^a^** | 7.0 ± 6.0 | 6.9 ± 6.2 | 0.651 |
| **Lymphovascular invasion** |  |  |  |
| Positive | 70(66.0) | 55(51.9) | 0.036 |
| Negative | 36(34.0) | 51(48.1) |  |
| **Tumor differentiation** |  |  |  |
| Well differentiated | 23(21.7) | 33(31.1) | 0.019 |
| Moderately differentiated | 49(46.2) | 56(52.8) |  |
| Poor differentiated | 34(32.1) | 17(16.1) |  |
| **Histological type** |  |  |  |
| Adenocarcinoma | 50(47.2) | 64(60.4) | 0.026 |
| Mucinous adenocarcinoma | 37(34.9) | 35(33.0) |  |
| Signet-ring cell carcinoma | 19(17.9) | 7(6.6) |  |

a: Mean ± Standard Deviation

**Table S3. Logistic regression model for risk prediction.**

|  | Univariable Analysis | | Multivariable Analysis | |
| --- | --- | --- | --- | --- |
| Variable | HR (95% CI) | *p* value | HR (95% CI) | *p* value |
| Mutation | 1.35(1.01-3.44) | 0.01 | 1.26(1.21-4.12) | 0.02 |
| Tumor differentiation | 1.81(1.45-6.78) | 0.03 | 1.78(1.21-5.41) | 0.04 |
| Histological type | 2.46(2.01-7.11) | 0.04 | 2.33(2.12-6.39) | 0.04 |
