# Supplementary material for: Somatic mutation correlation with lymph node metastasis and prognosis in T1/2 stage colorectal cancer patients: A propensity score matching analysis
Source: Clin Transl Med. 2025 Jan 7;15(1):e70179. doi: 10.1002/ctm2.70179 (PMC11705726; doi:10.1002/ctm2.70179)
Supplement: Supplementary file 2 — Supporting Information [file CTM2-15-e70179-s001.docx]

**Supplementary materials and methods**

*Inclusion and Exclusion Criteria*

All procedures involving human participants in this study adhered to ethical standards set by institutional and/or national research committees, as well as the 1964 Helsinki Declaration and its later amendments or similar ethical standards. This cohort study has been reported in line with the STROCSS criteria[[30](#_ENREF_30)] and was approved by institutional review boards of the First Affiliated Hospital of Nanjing Medical University. Informed consent was obtained from all patients. All the clinicopathological and molecular characteristics were detected through postoperative tumor tissues after complete resection.

Inclusion Criteria: Patients included in this study were diagnosed with colorectal cancer and received treatment at the Colorectal Center between January 2015 and February 2019. Pathological confirmation of malignant colorectal tumors was required, and the T stage was confirmed with T1 or T2. However, in the present study, all included specimens were obtained via colonoscopy biopsy prior to surgical intervention. Exclusion Criteria: patients with recurrent colorectal cancer, patients with multiple primary tumors, patients received local rection without lymph node dissection and patients with distant metastasis were excluded from the study. The clinical, pathological, and molecular variables, including patient age, gender, tumor stage, differentiation, and molecular testing, were collected for analysis. The patient selection process is illustrated in **Figure 1**.

*MSI Testing*

All patients underwent microsatellite instability (MSI) testing using a method that primarily employed multiplex fluorescent polymerase chain reaction (PCR) combined with capillary electrophoresis. This method involved PCR amplification of specific microsatellite sequences, followed by capillary electrophoresis to compare the differences in microsatellite sequence lengths between tumor tissue and normal tissue, determining the presence of MSI at specific loci. Genomic DNA was extracted from formalin-fixed paraffin-embedded (FFPE) tissue. The MSI testing kit (AmoyDx 8.0627301X024G) was used to assess five consistent mononucleotide repeat microsatellite markers (NR-24, BAT-25, CAT-25, BAT-26, MONO-27) in both tumor and normal tissue samples. Additionally, two pentanucleotide markers (Penta D and Penta E) were tested to confirm homogeneity between tumor and normal tissue. Capillary electrophoresis was performed using an ABI3500Dx genetic analyzer. The following criteria were used for MSI classification: (I) MSI-High (MSI-H) if two or more mononucleotide markers in tumor tissue had size variations of ≥3bp compared to normal tissue; (II) MSI-Low (MSI-L) if a single mononucleotide marker in tumor tissue exhibited a size variation of ≥3bp compared to normal tissue; (III) Microsatellite Stable (MSS) if there were no size variations of ≥3bp in mononucleotide markers in tumor tissue compared to normal tissue.

*DNA extraction and targeted NGS*

Formalin-fixed paraffin-embedded (FFPE) tumor samples obtained through surgical excision were collected. Genomic DNA from FFPE sections and whole blood control samples were extracted with the QIAamp DNA FFPE Tissue kit and DNeasy Blood and Tissue Kit (Qiagen, USA), respectively. Quantity and quality of the extracted DNA were evaluated by Qubit 3.0 fluorometer and Nanodrop 2000, respectively (Thermo Fisher Scientific).

Library preparations were performed using KAPA Hyper Prep kit (KAPA Biosystems) following manufacturer’s protocol. Hybridization-based target enrichment was carried out using the GeneseeqPrime® pan-cancer gene panel with xGen Lockdown Hybridization and Wash Reagents Kit (Integrated DNA Technologies). Captured libraries by Dynabeads M-270 (Life Technologies) were amplified in KAPA HiFi HotStart ReadyMix (KAPA Biosystems) and quantified by qPCR using KAPA Library Quantification Kit (KAPA Biosystems). The target-enriched library was then sequenced on the HiSeq4000 NGS platform (Illumina) following the manufacturer’s instructions.

*Sequence alignment and data processing*

Trimmomatic was used for FASTQ file quality control. Leading/ trailing low quality (below 20) or N bases were removed. The sequencing data was aligned to the reference Human Genome (hg19) using Burrows-Wheeler Aligner (BWA-mem, v0.7.12). Alignment results underwent de-duplication by Sambamba. Base quality recalibration and indel realignment were processed by Genome Analysis Toolkit (GATK 3.4.0). VarScan2 was employed for calling single-nucleotide variations (SNVs) and insertion/deletions (INDELs) which were identified with a minimum variant allele frequency threshold set at 0.01 and p value threshold for calling variants set at 0.05 to generate Variant Call Format files. All SNVs/indels were annotated with ANNOVAR. The sequencing assay has been validated in compliance with college of American pathologists (CAP) and clinical laboratory improvement amendments (CLIA) with a limit of detection of 1% VAF for tissue. Genomic fusions were identified by FACTERA with default parameters.

For mutation, single-nucleotide variants (SNVs) and indels were identified using VarScan2, with a minimum variant allele frequency at 0.1%. SNVs and indels were further filtered with the following parameters: (1) minimum mean dedup depth = 600X; (2) minimum base quality=15, (3) minimum variant supporting reads=3, (4) variant supporting reads mapped to both strands, (5) strand bias no greater than 10%, (6) if present in >1% population frequency in the 1000 g or ExAC database and (7) through an internally collected list of recurrent sequencing errors using a normal pool of 100 samples. ANNOVAR was used to annotate mutations by variant type [[31](#_ENREF_31)], dbSNP ID, clinical significance, and protein impact prediction using SIFT and PolyPhen[[32](#_ENREF_32)]. Germline mutations were filtered out by comparing them with patients' whole blood controls. For MRD, MRD positivity was defined by accessing the presence of one or more mutations identified in the matched tumor sample in ctDNA; In addition, for ctDNA variants not present in the corresponding primary tumor, samples were defined MRD positively if the following criteria were met,(i)ctDNA maximum somatic allele frequency (MSAF)≥1% (ii)at least three variants or one pathogenic variant detected in ctDNA.

*Nomogram model*

Variables that achieved significance at p <0 .05 in univariate analysis of the correlation with lymph node metastasis in the development cohort were entered into the multivariate analysis using a logistic regression model. In brief, On the basis of the final regression analysis, a nomogram was constructed that incorporated the significant risk factors for predicting lymph node metastasis. A total score was calculated with the use of mutation status, lymphovascular invasion, tumor differentiation and histological type. The value of each of these variables was given a score on the point scale axis. A total score could be easily calculated by adding each single score and, by projecting the total score to the lower total point scale, we were able to estimate the probability of lymph node metastasis. The performance of the nomogram was assessed by discrimination and calibration. The discriminative ability of the model was determined by the area under the receiver operating characteristic curve, which ranged from 0.5 (no discrimination) to 1 (perfect discrimination). The calibration of the prediction model was performed by a visual calibration plot comparing the predicted and actual probability of lymph node metastasis. In addition, the nomogram was subjected to 1000 bootstrap resamples for internal validation to assess their predictive accuracies.

*Propensity Score Matching (PSM) Analysis*

In the context of population-based datasets, comparing T1/2 stage CRC patients with positive lymph nodes against those with negative lymph nodes introduces concerns of potential endogeneity bias, which may stem from inherent dissimilarities between the cohorts. To address this challenge, a rigorous PSM methodology was devised to achieve balanced comparative groups. A propensity score for each patient was derived through a logistic regression model, in which the binary outcome of interest was lymph node status (positive or negative) for T1/2 stage cancers. The predictive model encompassed a comprehensive set of independent variables: age, gender, tumor location, tumor diameter, carcinoembryonic antigen (CEA) expression levels, and T staging. These factors were chosen based on their potential associations with both the likelihood of having positive lymph nodes and the clinical outcomes under investigation. Following the estimation of these propensity scores, a meticulous matching process was employed. Participants with negative lymph nodes were paired with those exhibiting positive lymph nodes using the nearest neighbor matching technique, with a stringent caliper width of 0.01 (1%) on the propensity score scale spanning from 0 to 1.

*Statistical Analysis*

Statistical analysis was performed using SPSS version 26.0 (IBM) and R version 4.0.4 (R Project for Statistical Computing) software. Two-tailed p-values less than 0.05 were considered statistically significant. Continuous variables were expressed as means (SD) or medians (IQR) based on data distribution, while categorical data were presented as numbers or percentages. For comparisons of continuous variables between groups, t-tests were used when data followed a normal distribution; otherwise, the Mann-Whitney U non-parametric test was employed. The comparison of categorical variables was conducted using the chi-squared test or Fisher's exact test. Additionally, based on NGS results, we selected mutations present in at least 5% of patients for survival staging, ensuring a minimum presence in 10 patients per group to mitigate bias and statistical uncertainty by guaranteeing sufficient events in each cohort. For survival analysis, Kaplan-Meier curves depicted survival outcomes, with log-rank tests comparing survival rates by mutation status post adjustment for multiple comparisons. Overall survival (OS) was measured from the end of systemic therapy until patient death.
